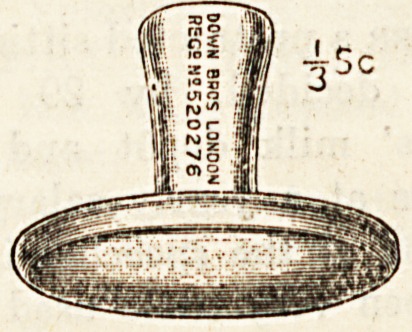# New Appliances & Things Medical

**Published:** 1909-02-20

**Authors:** 


### NEW APPLIANCES & THINGS MEDICAL.

[We shall be glad to receive at our Office, 28 & 29 Southampton Street, Strand, London, W.C., from the manufacturers, specimens of all new preparations and appliances.]

### THE GLASS OINTMENT APPLICATOR (REGISTERED).

Mr. James Donald, M.R.C.S., L.S.A., Medical Officer, Kingston Infirmary, and Honorary Medical Officer, Victoria Hospital, Kingston-on-Thames, writes as follows: A long-felt need has been supplied by the introduction of a new glass ointment applicator designed by Miss J. A. Smith.

When irritating ointments, or those which stain the hands, have to be applied in cases of ringworm, scabies, psoriasis, eczema, and other specific diseases, the glass applicator will be found almost indispensable. It is made in two forms, with plain or ground glass surfaces, and can be easily cleansed and sterilised. The ointment applicators have been in use in this infirmary for some time, and I find that they answer their purpose exceedingly well. This applicator is made by Messrs. Down Brothers, Limited, 21 St. Thomas's Street, Borough, London, S.E.

## Figures and Tables

**Figure f1:**
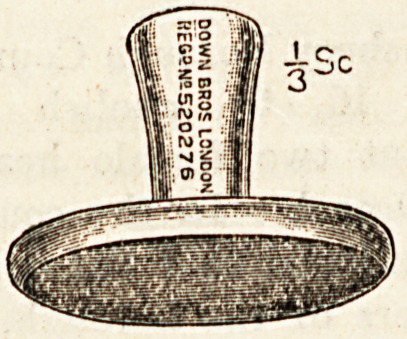


**Figure f2:**